# Spectrophotometric determination of olanzapine, fluoxetine HCL and its impurity using univariate and chemometrics methods reinforced by latin hypercube sampling: Validation and eco-friendliness assessments

**DOI:** 10.1186/s13065-024-01310-3

**Published:** 2024-10-17

**Authors:** Hussein N. Ghanem, Asmaa A. El-Zaher, Sally T. Mahmoud, Enas A. Taha

**Affiliations:** 1 Pharmaceutical Chemistry Department, Faculty of Pharmacy, Cairo University, Kasr El-Aini St., Cairo, 11562 Egypt; 2 Chemistry Department, Faculty of Pharmacy, October 6 University, 6 October City, Giza, 12585 Egypt

**Keywords:** Olanzapine/fluoxetine HCL combination, 4-(Trifluoromethyl) phenol toxic impurity, UV spectrophotometry, Chemometrics, Latin hypercube sampling technique, Comprehensive sustainability evaluations

## Abstract

Novel univariate and chemometrics-aided UV spectrophotometric methods were tailored to undergo the fundamentals of green and white analytical chemistry for the simultaneous estimation of a ternary mixture of olanzapine (OLA), fluoxetine HCL (FLU), and its toxic impurity 4-(Trifluoromethyl) phenol (FMP) without any prior separation. The dual-wavelength ratio spectrum univariate method was used to determine OLA and FLU in the presence of FMP in the range of (4–20) and (5–50) μg/ml, respectively. In compliance with the International Conference on Harmonization (ICH) standards, the technique was validated and established Remarkable accuracy (98–102%) and precision (< 2%) with limits of quantification (LOQs) of 0.432 and 2.002 μg/ml, respectively. Partial least squares (PLS) and artificial neural networks (ANNs) are chemometric methodologies that have advantages over the univariate method and use significant innovations employing Latin hypercube sampling (LHS), allowing the generation of a reliable validation set to guarantee the effectiveness and sustainability of these models. The concentration ranges used were (2–20), (2–20), and (5–50) μg/ml; for PLS, the LOQs were 0.602, 0.508, and 1.429 μg/ml, and the root mean square errors of prediction (RMSEPs) were 0.087, 0.048, and 0.159 for OLA, FMP, and FLU, respectively; and for ANNs, the LOQs were 0.551, 0.465, and 0.965 μg/ml, with RMSEPs of 0.056, 0.047, and 0.087 for OLA, FMP, and FLU, respectively. The developed methods yield a greener National Environmental Methods Index (NEMI) with an eco-scale assessment (ESA) score of 90 and a complementary Green Analytical Procedure Index (complex GAPI) in quadrants with an analytical greenness metric (AGREE) score of 0.8. The Red‒Green–Blue 12 algorithm (RGB 12) scored 88.9, outperforming on reported methods and demonstrating widespread practical and environmental approval. Statistical analysis revealed no noteworthy differences (P > 0.05) among the proposed and published techniques. Both pure powders and pharmaceutical capsules were analyzed via these methods.

**Supplementary Information:**

The online version contains supplementary material available at 10.1186/s13065-024-01310-3.

## Introduction

Researchers face a major challenge in achieving a balance between the effectiveness of analytical methods and their environmental sustainability (referred to as “greenness”), in addition to economic and practical aspects, which regularly contradict each other [1]. Recently, research societies have highlighted the integration of the principles of green analytical chemistry (GAC) and white analytical chemistry (WAC) into their research workflows [2, 3].

Multiple approaches have been employed to determine the eco-friendliness of analytical techniques with respect to the 12 GAC principles. These tools incorporate the National Environmental Method Index (NEMI) [4] Eco Scale Assessment (ESA) [5], Complementary Green Analytical Procedure Index (Complex GAPI) [6], and Analytical Greenness Metric (AGREE) [7]. All these implements aim to give a score or graphical output on the basis of particular standards of the environmental friendliness of the analytical methods under development [8, 9].

Furthermore, many algorithms have been used to assess the whiteness of these approaches, including multiple-criteria decision analysis (MCDA), HEXAGON, RGB, and the preferred red‒green–blue RGB 12 algorithm [10–13], because of its ease of use and user-friendliness. Chromatographic methods face challenges in meeting the criteria of GAC and WAC because of the demand for a large volume of hazardous organic solvents, intricate sample preparation techniques, excessive energy usage, and the utilization of expensive, complex equipment [14, 15].

As a result, there is still a great need to utilize simple, long-lasting, environmentally friendly, and affordable analytical methods that adhere to both GAC and WAC principles.

**Fig. 1:**
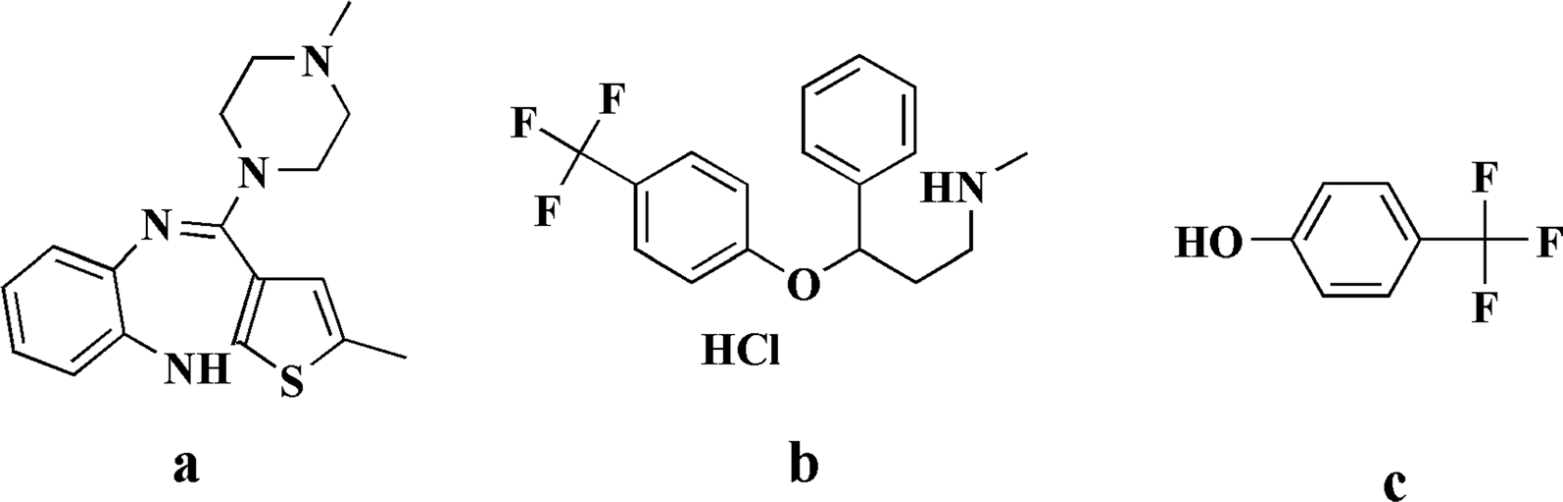
Displays the chemical structures of olanzapine **a**, fluoxetine HCL **b**, and 4–(Trifluoromethyl) phenol **c**

The FDA has approved olanzapine (OLA), an atypical antipsychotic drug used to treat bipolar disorder and schizophrenia; its chemical nomenclature is 2-methyl-4-(4-methyl-1-piperazinyl)-10H-thieno (2,3-b) (1,5) benzodiazepine (Fig. 1a) [16].

Fluoxetine HCL (FLU) belongs to the antidepressant class of selective serotonin reuptake inhibitors (SSRIs). Its chemical name is N-methyl-3-phenyl-3-[4-(trifluoromethyl) phenoxy] propane-1-amine (Fig. 1b) [16].

4-(Trifluoromethyl) phenol (FMP) is a toxic impurity of FLU that has respiratory tract irritation and serious eye damage. It belongs to the class of (trifluoromethyl)benzenes and is similar to p-cresol (Fig. 1c) [17].

Moreover, current spectroscopic techniques are typically used to analyze either OLA or FLU individually [18, 19], or their binary mixtures [16, 20–22]. There is no published spectroscopic method for assessing the ternary mixture of OLA, FLU, and FMP. Additionally, these methods rely on solvents that are not environmentally friendly, which goes against the principles of sustainability. Chromatographic techniques have also been published for these substances, but they do not fully adhere to the principles of GAC and WAC, as we previously discussed their drawbacks [22–26].

To study these challenges, the present investigation demonstrated that UV spectrophotometric techniques employing environmentally friendly solvents are reasonable options because of their ease of use, minimal solvent usage, simplicity, sensitivity, specificity, stability, repeatability, and environmental sustainability [27–30]. Various greenness and environmental impact assessments were conducted to confirm that our proposed methods offer superior environmental sustainability compared to the reported chromatographic methods. Three methods were developed as novel approaches for determining a ternary mixture of OLA, FLU, and FMP without prior separation. These methods include the univariate dual-wavelength ratio spectrum method and chemometrics-assisted techniques such as artificial neural networks (ANNs) and partial least squares (PLS). Chemometric methods offer benefits such as shorter analysis times and stages, detection of lower concentrations within the linear range, and quantification of impurity concentrations.

However, many current chemometric studies rely primarily on random data selection to create training and validation portions [31], while this method is straightforward, it risks generating validation sets that do not adequately encompass the entire range of samples. Consequently, this approach may result in biased model accuracy, which contradicts the objectives of dependability and resource optimization. To address this substantial obstacle, this research utilizes a statistical approach called Latin hypercube sampling (LHS) to design representative validation sets systematically [32, 33]. LHS divides each modeled variable's range into equally likely segments and guarantees that each segment is encompassed within the resulting validation dataset. This approach attains exceptional balance and coverage, enabling a comprehensive and impartial evaluation of the chemometric model's predictive abilities and enhancing reliability with minimal validation sample usage. In addition to reducing material consumption and waste, LHS strengthens sustainability initiatives. Additionally, it helps prevent erroneous assessments of model accuracy that could impede quality control whenever untrustworthy chemometric tools are employed. Given its benefits, LHS is highly suitable for promoting environmentally friendly and dependable chemometric techniques in pharmaceutical analysis with enhanced sample efficiency.

The primary aim of this research is to optimize new, straightforward, valid, and sensitive univariate and chemometric-assisted UV methods by integrating LHS as a critical component in chemometric validation. These methods are used for the determination of ternary mixtures without the need for preliminary isolation, which aligns with the fundamentals of GAC and WAC. To evaluate the proposed approaches, various evaluation tools, including NEMI, ESA, Complex GAPI, AGREE, and RGB 12, have been employed to compare their performance with that of previously published methods With regard to environmental sustainability.

## Experimental

### Chemicals and reagents

OLA and FLU were supplied by October Pharma Co., Giza, Egypt, with verified purities of 99.25% and 96.65%, respectively, and FMP was acquired from Sigma‒Aldrich with a verified purity of 97%. Ethanol high-performance liquid chromatography (HPLC) products were acquired from Sigma‒Aldrich. Flunazapine^®^ capsules B.N.20622, manufactured by Delta Pharmaceutical Company in Cairo, Egypt, including 12 mg OLA and 25 mg FLU per capsule, were acquired from a community pharmacy.

### Instrumentation and software

A UV‒1601 PC Shimadzu UV‒vis spectrophotometer equipped with UV‒120 probe software was utilized. An ultrasonic sonicator and Shimadzu electronic weighing scale were also used. PLS, ANNs and LHS were performed in MATLAB^®^ R2013b (8.2.0.701) via the PLS toolbox 2.1. AGREE and Complex GAPI software tools were used for eco-friendliness assessment, and Excel was used for statistical analysis.

### Standard stock and working solutions

Standard stock solutions of OLA, FMP, and FLU were made individually at 1 mg/mL in ethanol. These solutions were subsequently diluted to make the standard working solutions 50, 50, and 125 μg/ml, respectively, via the same solvent.

### Dual-wavelength ratio spectrum method

Various portions of OLA and FLU standard working solutions were put into 10 mL volumetric flasks and diluted to the mark with ethanol to perform calibrations of (4–20) and (5–50) μg/ml, respectively. The absorption spectra (ranging from 200–400 nm) of these solutions were captured with ethanol as a reference blank and were saved on a computer. Lab-created mixtures of OLA, FLU, and FMP were prepared.

### Experimental design of the multivariate methods

Creating a meticulously planned framework for the experiment is essential to make sure that the gathering of data is relevant and representative. The calibration and validation sets were developed following the Brereton multilevel multifactor experimental design [34]. A design featuring three factors, each with five levels, was created for the determination of OLA, FMP, and FLU, resulting in 25 lab-created mixtures of the mentioned drugs (Table 1). The concentrations selected for every compound were determined according to their linear ranges: (2–20), (2–20), and (5–50) μg/ml. Moreover, the proportions of the two compounds found in their pharmaceutical formulations were considered. The validation set was generated via LHS from the provided design, ensuring a representative selection from the concentration range for dependable model validation. The design has numerous advantages, such as ease of use, affordability, minimal solvent consumption, time efficiency, and eco-friendliness. Various portions of OLA, FMP, and FLU were transferred from their working solutions to volumetric flasks of 10 ml to create diverse concentrations of these lab-created mixtures. Owing to high noise levels and diminished analyte signals, the absorption spectra were captured within the range of 210–310 nm at 0.1 nm intervals for PLS and at 1 nm for ANNs.

**Table 1:** 3-factors, 5-levels design based on the Brereton multilevel multifactor experiment

Mix	OLA	FMP	FLU
1	6	6	15
2*	6	2	5
3	2	10	5
4	2	4	25
5	10	10	10
6	4	6	25
7*	10	4	15
8	6	4	10
9*	4	8	10
10*	4	10	20
11	8	8	25
12	10	6	20
13*	8	10	15
14*	6	10	25
15*	10	2	25
16	10	8	5
17	2	2	20
18	8	6	5
19	2	8	15
20	6	8	20
21	8	4	20
22	8	2	10
23	4	4	5
24*	2	6	10
25	4	2	15

^*^Validation set

To evaluate the final chemometric models that were developed, several analytical effectiveness measures were computed [35]. The RMSEC, SEC, and RMSECV, which represent the root mean square error of calibration, standard error of calibration, and root mean square error of cross-validation, respectively, were computed for the calibration set.

With respect to the validation set, the root mean square error of prediction (RMSEP) was used to evaluate the overall ability of the model to generalize. The relative percentage error of the prediction RE (%) reflects the accuracy of the predictions. Moreover, the bias-corrected mean square error of prediction (BCRMSEP) was used to assess the precision and variability of predictions on new samples.

The following equation is used to compute the RMSECV, RMSEP, and RMSEC:

$$\text{RMSE}=\sqrt{\frac{\sum_{\text{i}=1}^{\text{n}} (\text{xi}-\widehat{\text{x}}\text{i}{)}^{2}}{\text{n}}}$$

The following equations were utilized to determine the remaining figures of merit:

$$\text{Bias }=\frac{\sum_{\text{i}=1}^{\text{n}} (\text{xi}-\widehat{\text{x}}\text{i})}{\text{n}}$$

$$\text{SEC}=\sqrt{\frac{\sum_{\text{i}=1}^{\text{n}} (\text{xi}-\widehat{\text{x}}\text{i}-\text{ bias }{)}^{2}}{\text{n}-1}}$$

$${\text{RE(\% )}}\, = \,100\,\sqrt {\frac{{\sum {_{{\text{i = 1}}}^{{\text{n}}} \,\left( {{\text{xi}} - \widehat{{\text{x}}}{\text{i}}} \right)^{2} } }}{{\sum {_{{\text{i = 1}}}^{{\text{n}}} \,{\text{xi}}^{2} } }}}$$

$$\text{BCRMSEP}=\frac{\sum_{\text{i}=1}^{\text{n}} (\text{xi}-\widehat{\text{x}}\text{i}{)}^{2}}{\text{n}}-(\text{ bias }{)}^{2}$$

where $xi$ is the known analyte concentration in sample $i$, $\widehat{x}i$ is the anticipated concentration, and $n$ is the overall number of samples in the validation set.

### Pharmaceutical applications

We carefully emptied and weighed ten Flunazapine^®^ capsules. A certain amount of the powder equal to 12 mg of OLA and 25 mg of FLU was perfectly weighed and dissolved in a 100 mL volumetric flask utilizing ethanol and ultrasonication for 5 min. Then, the mixture was filled with ethanol to the mark, the mixture was filtered, and a 10 mL volumetric flask was partially filled with 0.5 mL of the filtrate. The residual volume was then filled with ethanol to the mark, resulting in end concentrations of 6 μg/ml for OLA and 12.5 μg/ml for FLU. The absorption spectra were recorded using ethanol as the blank.

## Results and discussion

### Dual-wavelength ratio spectrum method

**Fig. 2:**
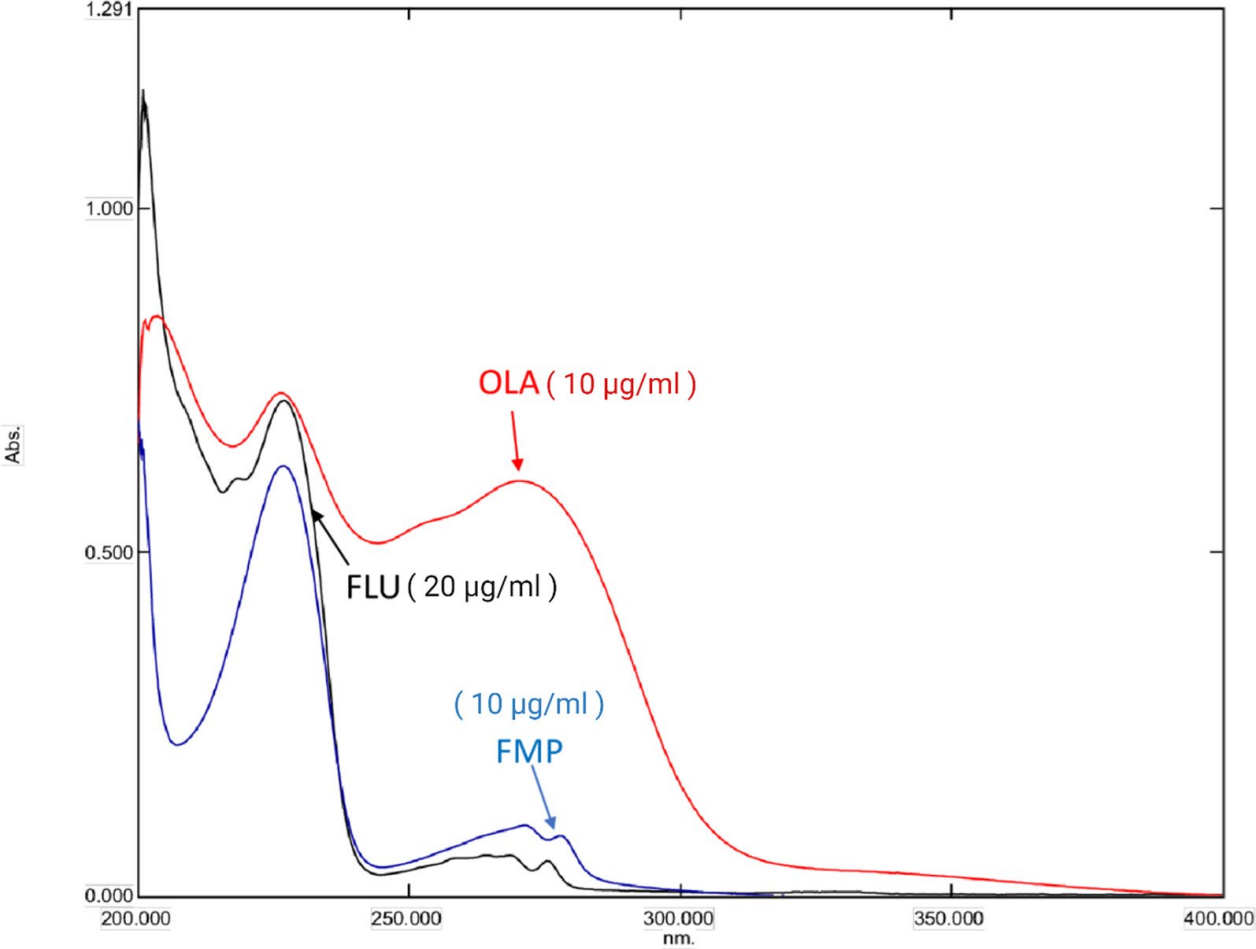
OLA, FMP, and FLU zero-order absorption spectra, displaying strong overlap

**Fig. 3:**
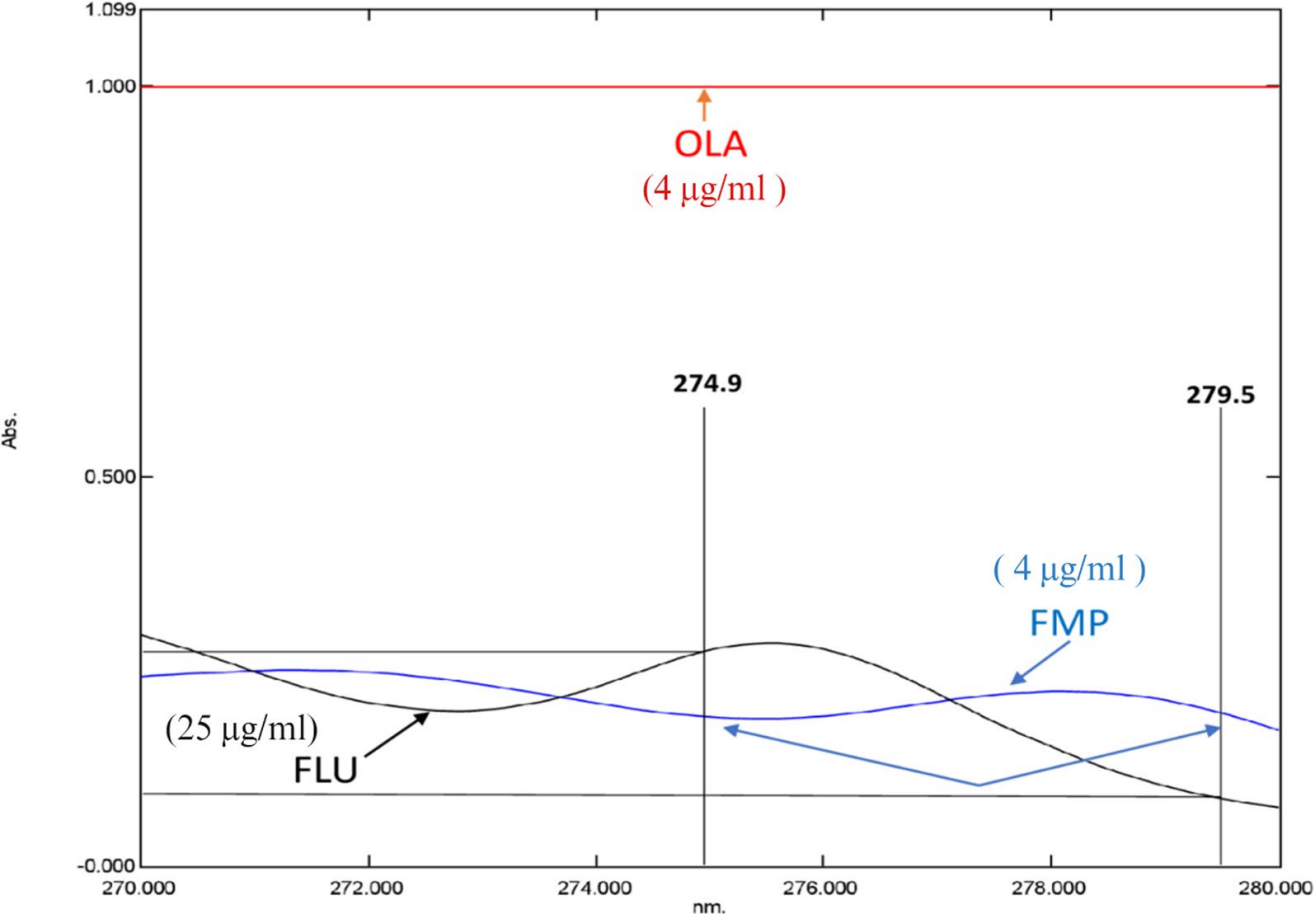
Ratio spectra of OLA, FMP, and FLU divided by OLA (4 µg/ml) showing the wavelengths selected for determination of FLU

**Fig. 4:**
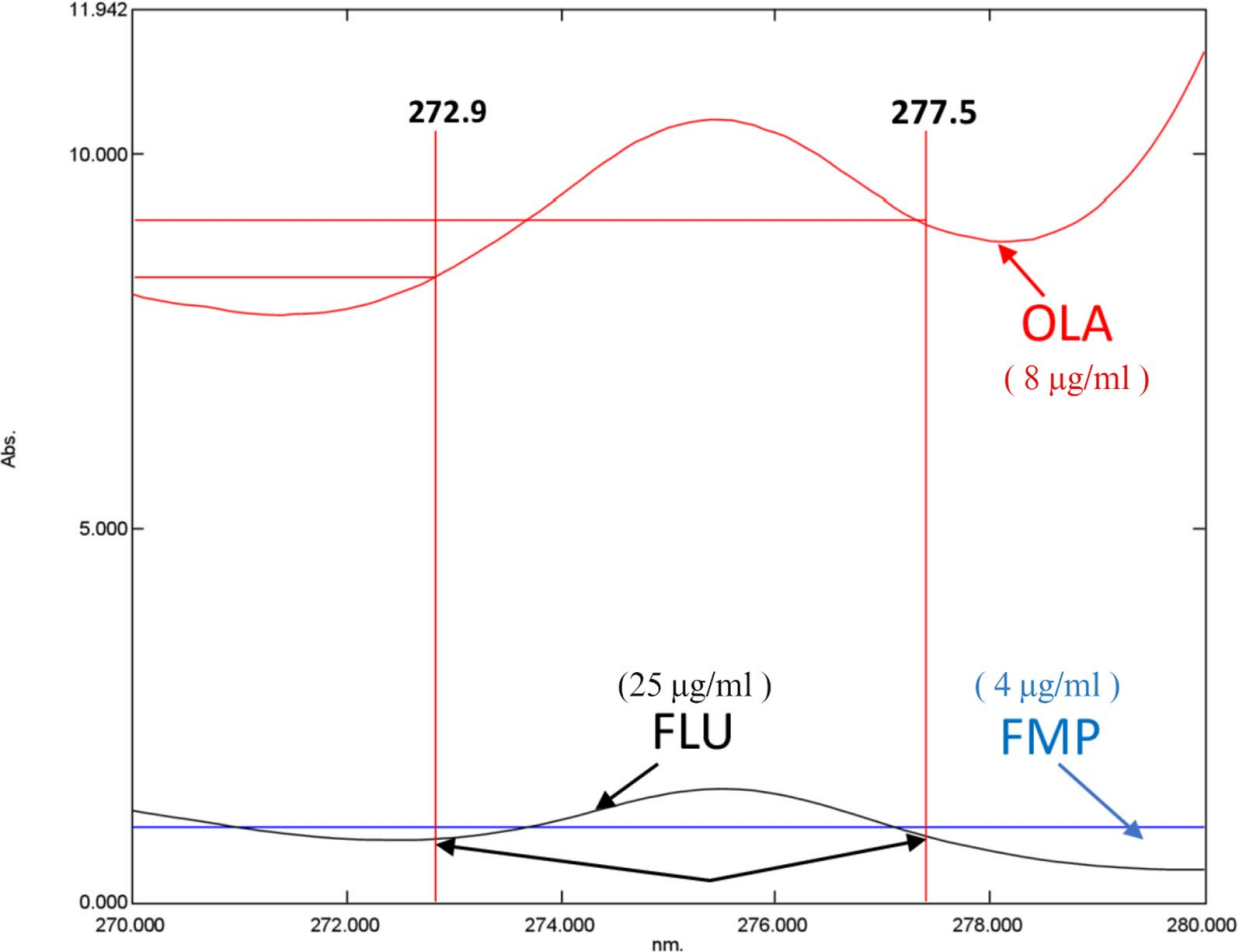
Ratio spectra of OLA, FMP, and FLU divided by FMP (4 µg/ml) showing the wavelengths selected for determination of OLA

The suggested approach begins by examining the zero-order spectra of OLA, FLU, and FMP (Fig. 2). Afterward, various divisor concentrations were tested. Care should be taken while choosing the divisors to achieve the ideal balance between maximum sensitivity and minimum noise to determine OLA and FLU simultaneously; in the presence of FMP, the stored absorption spectra were divided by FMP (4 μg/ml) as a divisor to determine OLA and divided by OLA (4 μg/ml) to determine FLU. The difference in the ratio spectra’s peak amplitudes was calculated for OLA (at 272.9 and 277.5 nm) (wavelengths at which FLU displays an identical amplitude) and for FLU (at 274.9 and 279.5 nm) (wavelengths at which FMP displays an identical amplitude) (Figs. 3, 4). Calibration curves were created using the difference in peak amplitudes [36] Calibration curves were determined to exhibit linearity across the concentration ranges of (4–20) and (5–50) μg/ml for OLA and FLU, respectively (Figs. 1S, 2S). The limits of quantification (LOQs) and limits of detection (LODs) were determined depending on the standard deviation of the intercept and were computed as follows:

LOD = 3.3 × SD of the intercept/slope coefficient.

LOQ = 10 × SD of the intercept/slope coefficient.

The International Conference for Harmonization (ICH) guidelines were followed when the approach was validated. [37] (Table 2).

**Table 2:** Dual wavelength ratio spectrum method according to International Conference for Harmonisation (ICH) guidelines and pharmaceutical application

Parameters	OLA	FLU
Accuracy ± RSD%^a^	100.72 ± 0.252	99.309 ± 1.277
Regression equation	y = 0.0677x + 0.045	y = 0.0075x—0.002
Correlation coefficient R^2^	1	0.999
Range (μg/ml)	4–20	5–50
Intraday precision RSD%^b^	1.229	0.805
Intraday precision RSD%^c^	1.149	1.001
Robustness RSD%^d^	0.646	1.179
LOD (μg/ml)	0.143	0.661
LOQ (μg/ml)	0.432	2.002
Pharmaceutical^e^ ± SD	99.457 ± 1.218	101.368 ± .575

^a^mean of five determinations

^b^Repeatability (n = 9), a mean of three concentrations of OLA (8,10,15 µg/ml), FLU (10,20,35 µg/ml) repeated three times within the day (intra-daily)

^c^The inter-daily precision (n = 9), a mean of three concentrations of OLA (6,10,15 µg/ml), FLU (10,15,25 µg/ml) repeated three times on three successive days

^d^Robustness (slight modification to the method) (n = 6), a mean of three concentrations of OLA (4,15 µg/ml), FLU (12.5, 20 µg/ml) repeated three times

^e^mean of three determinations

### Specificity

The selectivity was evaluated by analyzing several lab-created mixtures with concentrations of OLA, FMP, and FLU within the linearity range, promising results were achieved. Standard addition techniques were applied (Table 3).

**Table 3:** Results of lab created mixtures of OLA, FMP, and FLU by the suggested univariate method and applying of standard addition techniques

OLA (μg/ml)	R	FLU (μg/ml)	R	FMP (μg/ml)	R
6	101.969 ± 0.512	10	97.733 ± 0.965	4	–
6	99.312 ± 1.146	15	99.822 ± 1.463	6	–
8	99.556 ± 1.523	25	102.026 ± 0.852	8	–
8	101.218 ± 0.652	15	99.822 ± 1.846	10	–
Sd	1.464	Sd	1.956		

Std addition	R	Std addition	R
5	100.443 ± 1.203	6.5	98.457 ± 0.965
6	101.597 ± 1.186	13	100.222 ± 1.631
7	101.357 ± 0.532	18.5	99.737 ± 1.354
Sd	1.034	Sd	1.407

### Multivariate methods

Wavelengths falling between 310 and 400 nm were omitted because there were no absorbance values in this range. To avoid interference caused by noisy spectra, wavelengths below 210 nm were also excluded. Hence, the provided chemometric models, PLS and ANNs, utilized wavelengths ranging from 210 to 310 nm, Various interval values were tested, with RMSECV as the primary performance criterion. The optimization process found that 0.1 nm intervals produced the most reliable results for PLS, due to their high sensitivity to subtle spectral variations. In contrast, a 1 nm interval was optimal for ANNs, striking the best balance between computational efficiency and model accuracy. These intervals were selected for their ability to minimize the RMSEP error function, ensuring robust and accurate model predictions. Against ethanol as a blank a total of 17 combinations of the investigated drugs were determined for the calibration set. The remaining 8 combinations were chosen as the external validation set, utilizing the statistical method LHS. The calibration set’s absorbance and concentration matrices were employed in constructing these models via MATLAB^®^ R2013b and the PLS toolbox 2.1. These findings were subsequently validated via an external validation set.

### Validation set design

**Fig. 5:**
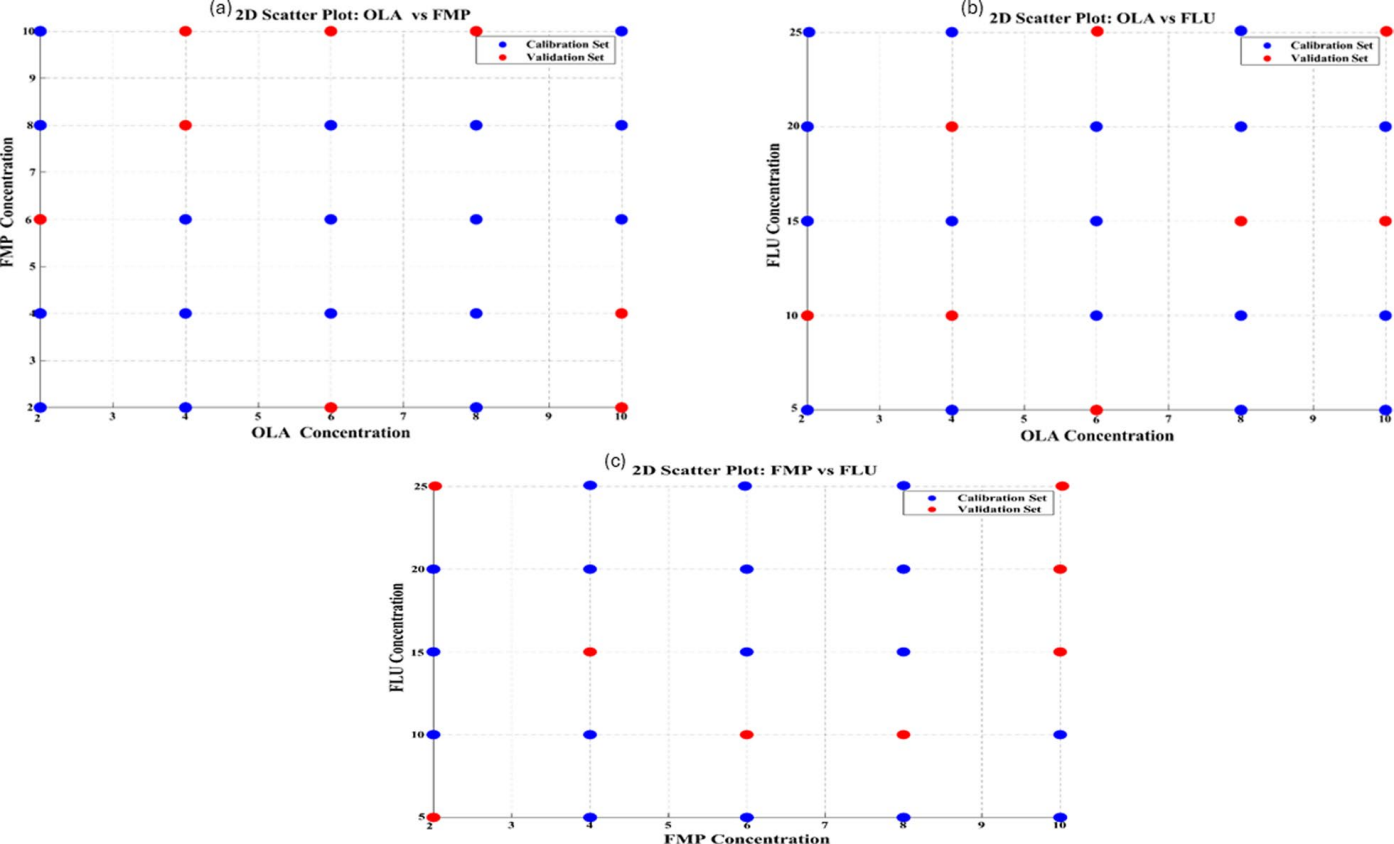
Latin Hypercube sampling design as ideal-Space Filling construction for the validation set. **a** 2D scatter diagram of OLA/FMP, **b** 2D scatter diagram of OLA/FLU, and **c** 2D scatter diagram of FMP/FLU

Creating a well-designed validation set was crucial for evaluating the predictive accuracy of the chemometric models across a wide array of analyte combinations. Random sampling carries the risk of incomplete coverage, leading to partial accuracy assessments. To address this issue, we employed a systematic approach by utilizing LHS, a statistically effective method for experimental design. LHS partitions the concentration range of every component into similar likelihood strata. We employed a perfect validation set size of 8 mixtures chosen via LHS, which determined one sample from every stratum to guarantee uniformity in all aspects of the concentration area. This is depicted in scatter plots, illustrating uniform scattering of the 8 validation samples throughout all analyte ranges (Fig. 5). Unlike random sampling, LHS provides better representativeness and coverage while using fewer samples, enhancing method efficiency and minimizing material consumption, waste, and expenses. This approach aligns closely with the essential concepts of creating sustainable analytical techniques.

### PLS

**Fig. 6:**
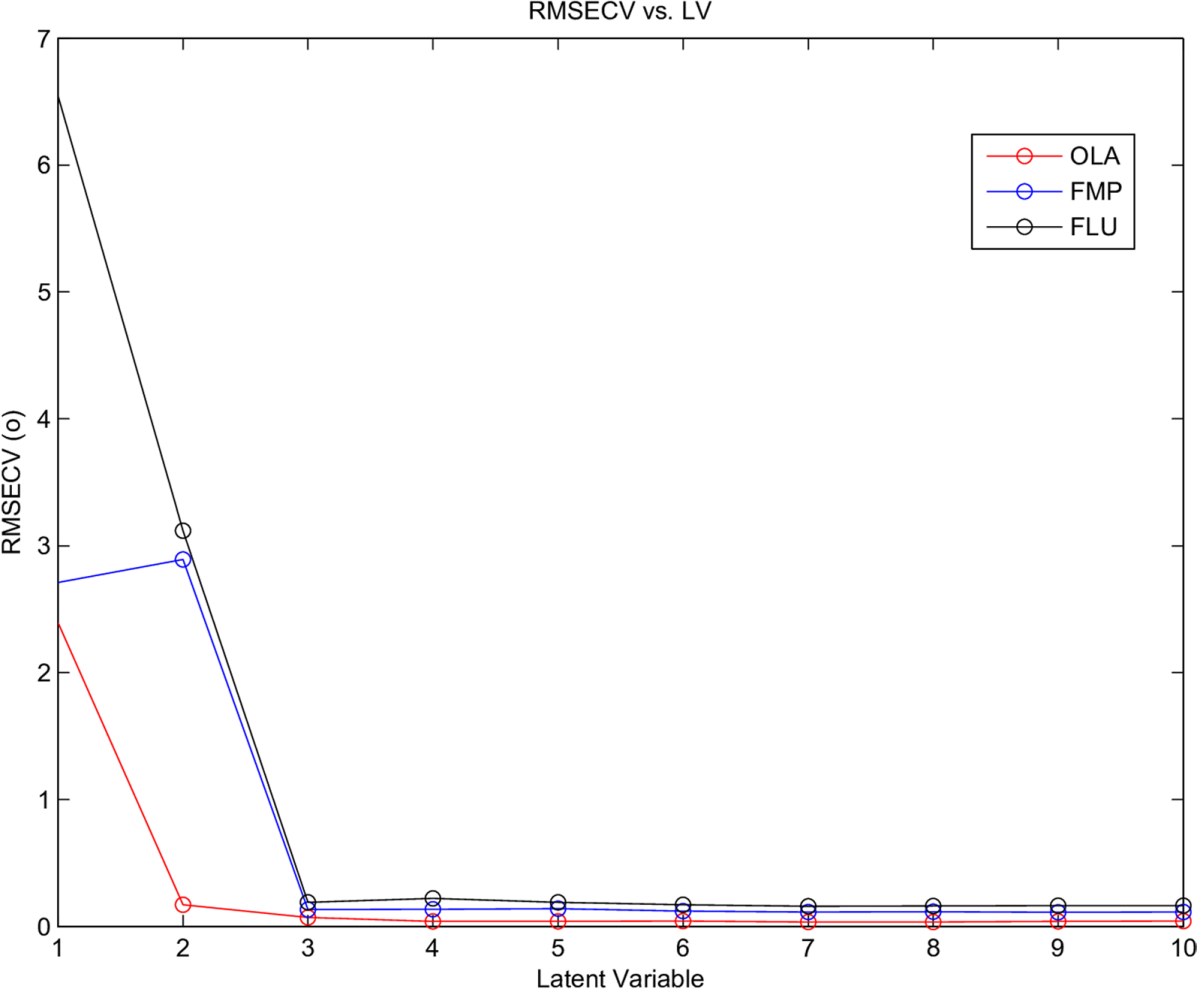
Latent variable against RMSECV Showing the Optimal latent variables selection was four

PLS is the predominant and most commonly employed chemometric technique for creating multivariate calibration sets [38–40]. To build the PLS model, we utilized MATLAB^®^ and PLS Toolbox 2.1. The cross-validation process, involving the exclusion of one sample at a time, was employed. Four latent variables (LVs) were deemed optimal, as determined by the standards established by Haaland and Thomas’ criteria [41]. The determination of the ideal number of LVs relies on the minimum RMSECV (Fig. 6).

Figures of Merit, such as RMSEC, SEC, RMSEP, RE (%), and BCRMSEP, were calculated to determine the performance of calibration and prediction (Table 4).

**Table 4:** Result of calibration, external validation set, and pharmaceutical application for multivariate methods

	PLS			ANNs		
	OLA	FMP	FLU	OLA	FMP	FLU
Calibration set						
Mean R	100	99.955	99.981	100.049	100.172	100.353
SD	0.764	1.644	1.232	0.606	1.138	0.973
RMSEC	0.026	0.081	0.142	0.048	0.052	0.181
SEC	0.083	0.052	0.225	0.055	0.055	0.097
Validation set						
Mean R	99.851	99.751	100.553	99.582	100.524	99.826
SD	1.521	1.253	0.989	1.36	0.59	0.883
RMSEP	0.087	0.048	0.159	0.056	0.047	0.087
RE%	1.278	0.658	0.934	0.821	0.645	0.508
BCRNSEP	0.006	0.002	0.016	0.002	0.002	0.007
LOD (μg/ml)	0.199	0.167	0.472	0.182	0.153	0.318
LOQ (μg/ml)	0.602	0.508	1.429	0.551	0.465	0.965
Pharmaceutical^a^	99.58		101.2	99.106		99.848
± SD	± .956		± .505	± .748		± .456

^a^Average of three determinations

### Anns

**Fig. 7:**
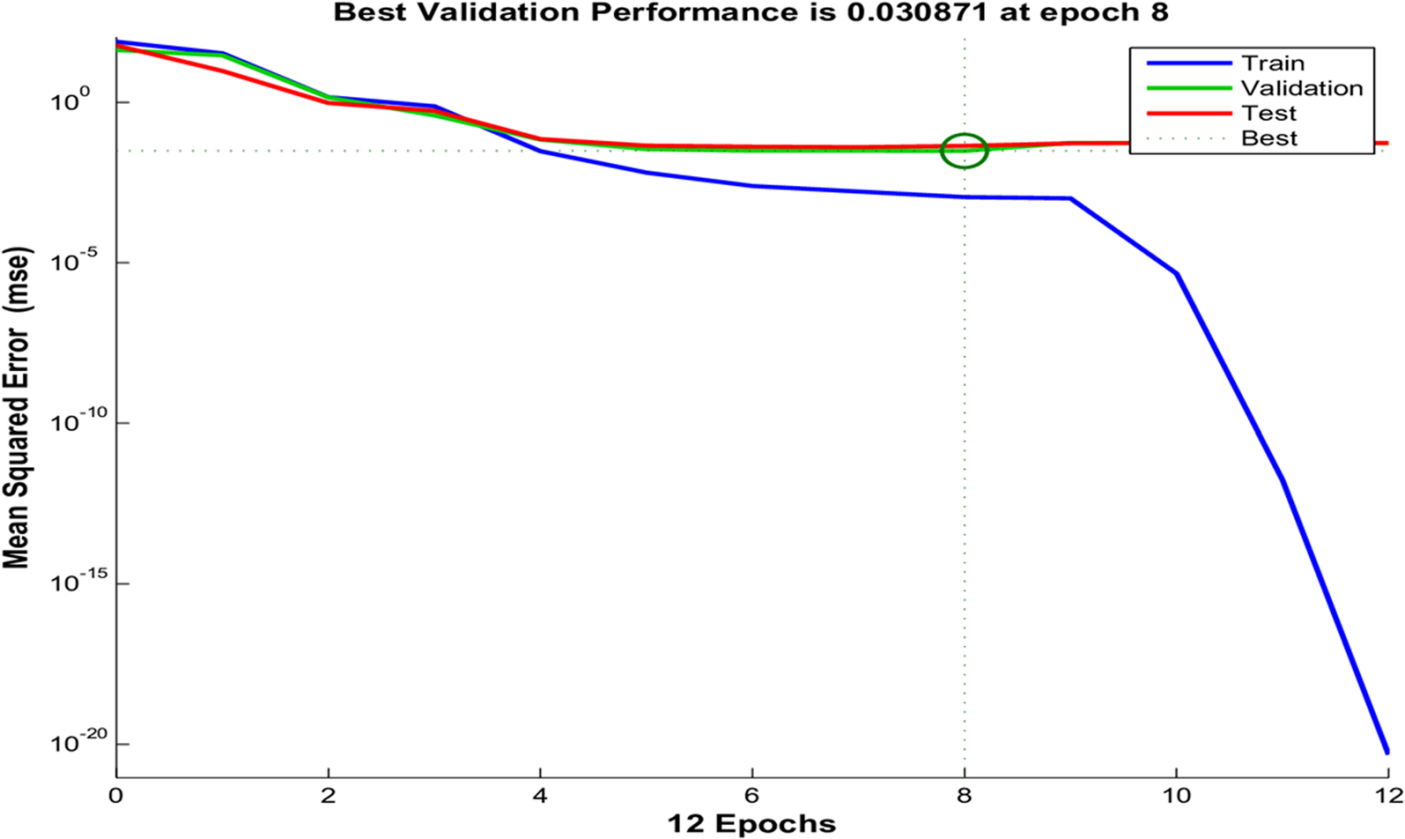
Number of Epochs versus MSE values for LM algorithm

**Fig. 8:**
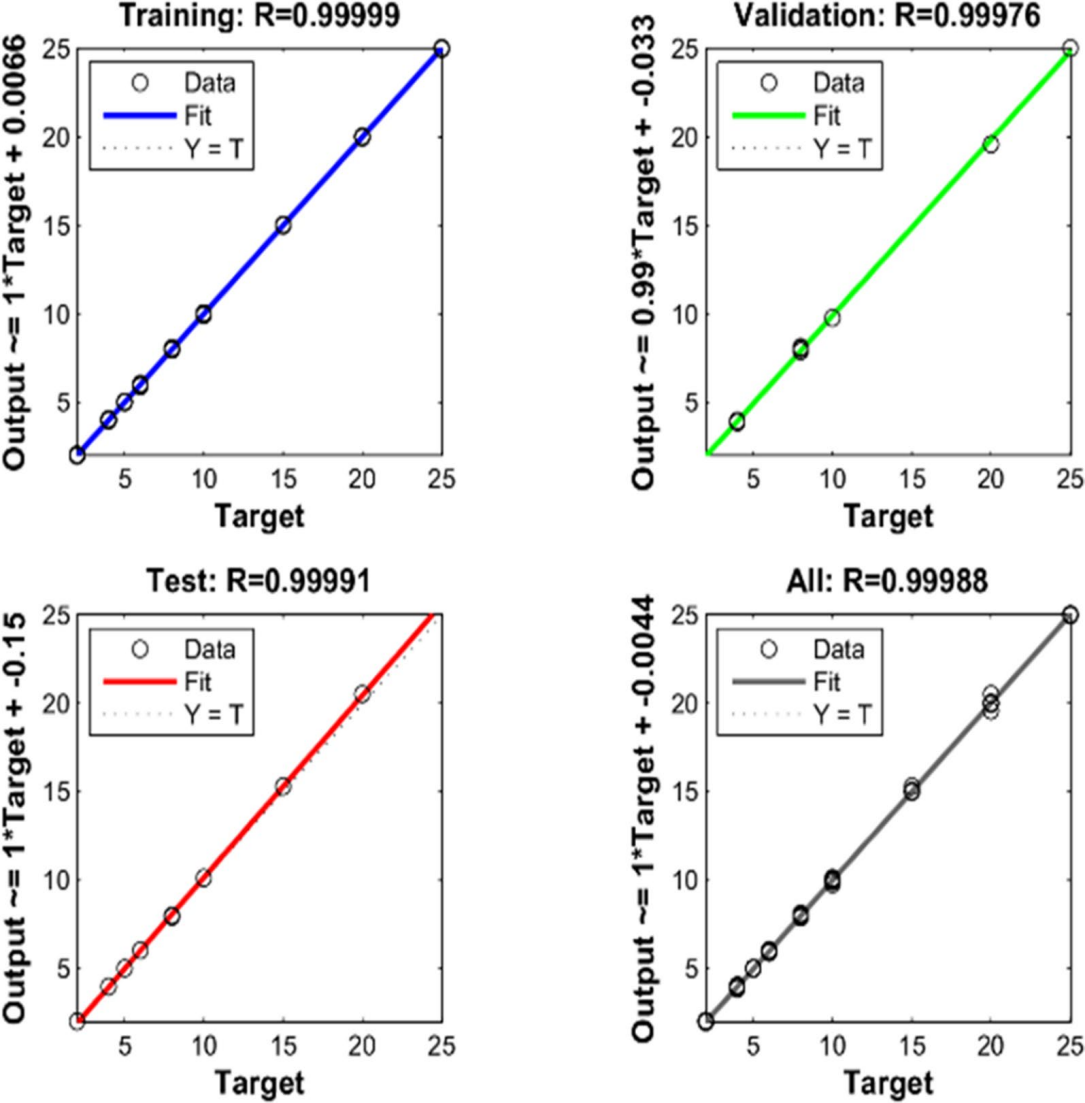
Diagrams of the LM algorithm for training, validation, and testing

A computing system that emulates how the human brain examines and processes data [42]. To improve the performance of a neural network, an iterative approach, the Levenberg–Marquardt (LM) algorithm, must be utilized to find the most effective neural network architecture. The error function RMSEP is utilized as a standard for concluding the process of learning (Figs. 7, 8). The ANNs comprise three layers: input, hidden, and output layers. In this configuration, 101 neuron data points were employed for the input layer. After experimentation, an optimal setup consisting of 5 neurons in the hidden layer was determined. Additionally, the output layer was designed with three neurons, one for each component. Numerous experiments were conducted to increase the performance of the model.

Figures of Merit, such as RMSEC, SEC, RMSEP, RE (%), and BCRMSEP, were calculated to determine the performance of calibration and prediction (Table 4).

### Evaluation of the greenness and whiteness profile of the method

#### Greenness assessment based on NEMI

*The* NEMI represents an earlier qualitative method in the realm of greenness evaluation and gives valuable insights into the determination of environmental friendliness. A circular diagram is split into four quadrants: corrosive, hazardous, persistent, bioaccumulative, and toxic (PBT), and waste (Fig. 3S). This circle is tinted green if specific requirements are fulfilled. These criteria encompass ensuring that the chemicals involved in the procedure are not classified as Persistent, Bioaccumulative, or Toxic (PBT) as per the EPA’s Toxic Release Inventory (TRI) Agency classification. The pH was verified to fall within a noncorrosive range (between 2 and 12). Waste production should be maintained below 50 g[4]. In our proposed approaches, we created NEMI pictograms. The greenness of our suggested approaches became evident, as it fulfilled all four NEMI criteria by having all four quadrants colored green, in contrast to previously reported chromatographic methods[23, 26] (Table 5).

**Table 5:**
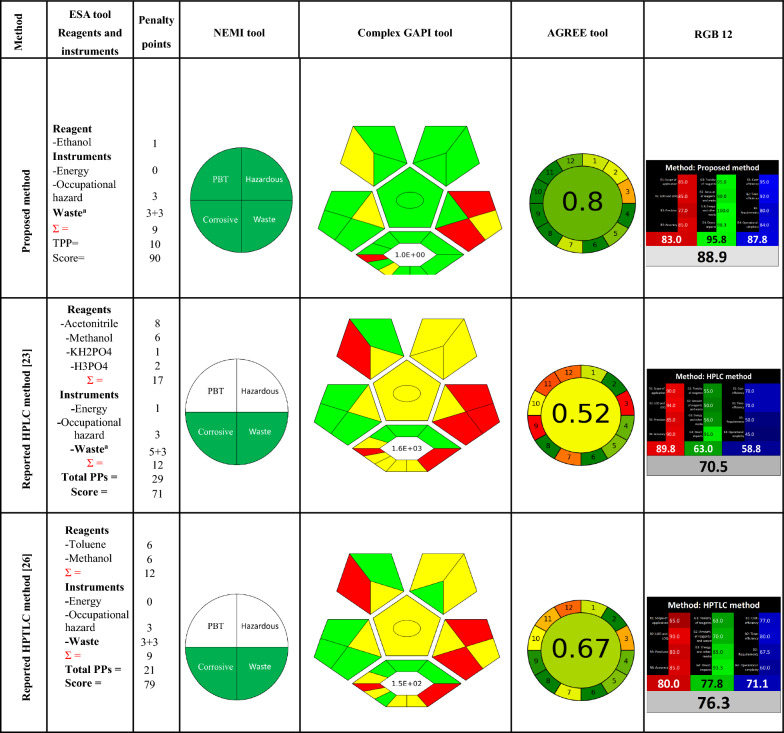
Comparison of the greenness and whiteness profiles of proposed and reported method using NEMI, ESA, Complex GAPI, AGREE and RGB 12 tool

^a^Run time × flow rate []

#### *Evaluation of greenness *via* ESA*

ESA (Eco Scale Assessment) is a newer and more sophisticated semiquantitative tool intended to evaluate the effects of a methodology on the environment. ESA involves deducting penalty points assigned for analytical process features that do not adhere to the 12 fundamentals of GAC (Fig. 4S). A greener analysis receives a greater score, approaching 100 [5]. The ESA scores for our proposed methods were detected. Notably, our suggested approaches exhibited remarkable greenness, as indicated by a high ESA score of 90 points, in contrast to previously reported chromatographic methods [23, 26] (Table 5).

#### Greenness evaluation via complex GAPI

More recently, a newer semiquantitative tool called ComplexGAPI has received significant attention, trust, and acceptance within the chemical society. This tool has simplified and improved the existing GAPI metric by introducing another hexagonal region into the initial GAPI graph (Fig. 5S). It relies on CHEM21 parameters that incorporate the various stages and procedures occurring prior to the overall analytical approach and the final analysis, which means that it can assess all steps of an analytical method, involving sample gathering, conveyance, preservation, storage, sample preparation, and preliminary procedures before the actual analysis. Notably, Complex GAPI uses shareware software for generating complex GAPI pictograms, making it user friendly. Interestingly, the produced pictogram transitions from green to yellow to red, enabling the assessment and quantification of every stage preceding the overall analytical methodology and concluding with the final analysis [6]. The methods outlined here are environmentally friendly, as indicated by the green pictograms and the E factor. The suggested approaches demonstrate a reduced E factor, equal to (1), indicating decreased waste generation, improved environmental impact, and increased sustainability. This demonstrates the advantage of the described methods in terms of eco-friendliness in contrast to previously reported chromatographic methods [23, 26] (Table 5).

#### *Greenness evaluation *via* AGREE*

AGREE is currently the most popular eco-friendliness assessment criterion. It is comprehensive, encompassing all 12 principles of GAC. It is also flexible, permits weighting, is presented in a user-friendly manner (resulting in a color-coded pictogram), and is easy to implement via readily available software. The input parameters incorporate the 12 essential principles, allowing for the assignment of different weights to enhance flexibility. These 12 input parameters are subsequently converted into a final score ranging from 0 to 1. The outcome is represented graphically, resembling a timepiece with a score and color in the center that reflects the final score. This score can range from dark green (= 1) to dark red (= 0) (Fig. 6S.)[7]. Before performing a comprehensive evaluation via multicolor diagrams, we initially documented essential information concerning the suggested methods and compared it to previously published methods regarding the 12 GAC parameters. The graphs illustrate the exceptional eco-friendliness of the suggested methods, with a score of 0.8, which is indicative of their superior green effect in contrast to previously reported chromatographic methods [23, 26] (Table 5).

#### Assessment of the whiteness

**Fig. 9:**
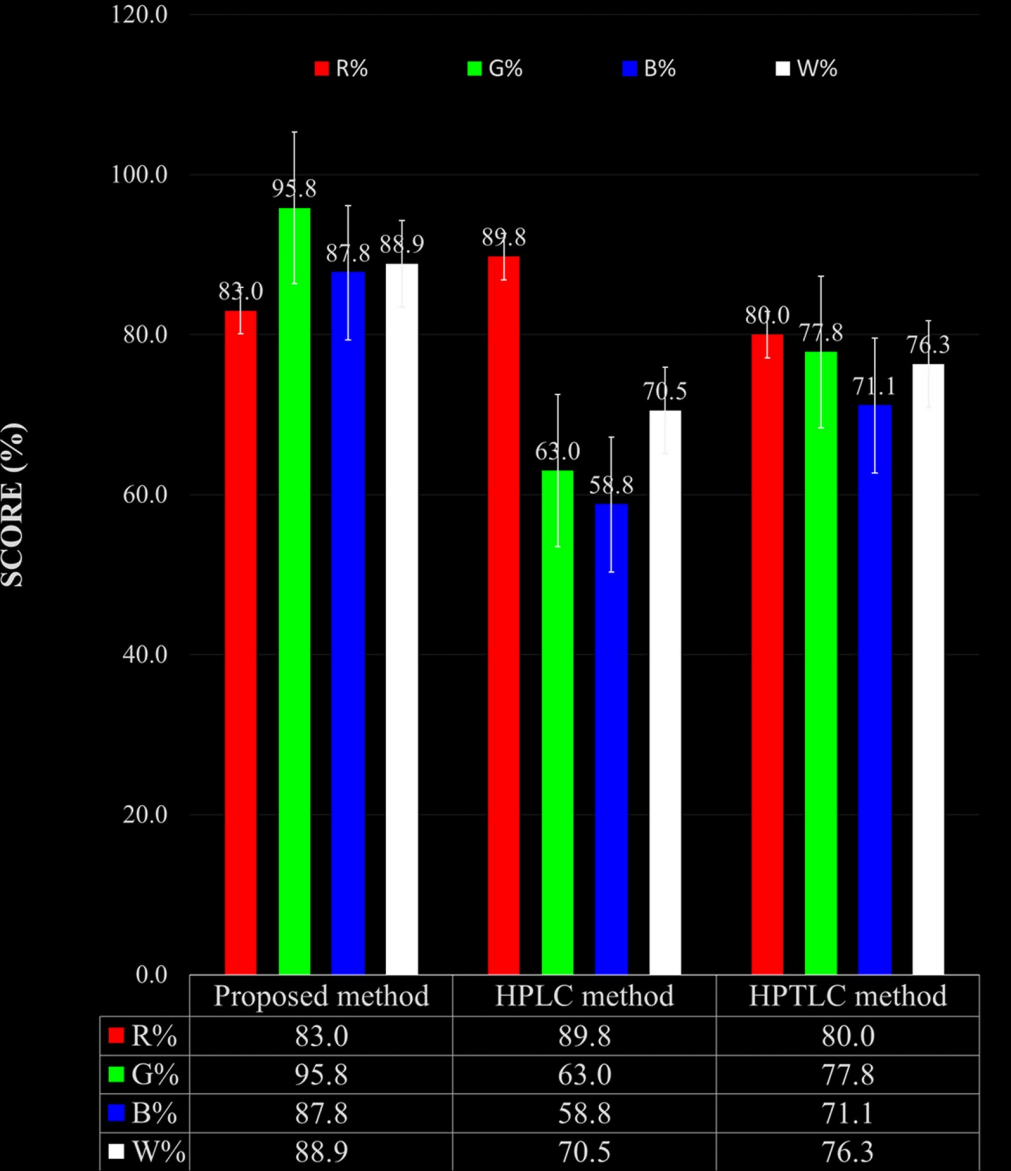
Whiteness of the proposed method compared to other published methods

The RGB 12 tool, which Pawe-Nowak and coauthors introduced in June 2021, is an easily adaptable quantitative tool for evaluating eco-friendliness. This tool provides a simple assessment of methods built upon the 12 WAC impacts and determines the degree of sustainability concerning eco-friendliness assessment. The RGB 12 algorithm includes 12 different algorithms subdivided into four classes: green, blue, and red. The green category (G1–G4) focuses on significant GAC parameters, such as toxicity, reagent and waste quantities, energy requirements, and impacts on people, animals, and genetic modifications. The red category (R1–R4) addresses validation factors, including applicability, accuracy, precision, LOD, and LOQ. The blue category (B1–B4) evaluates affordability, time effectiveness, and practical and economic factors. The overall “whiteness” value, which measures method compliance with WAC principles, is estimated by summing the scores across all three colors via the RGB 12 algorithm [13]. The suggested methods demonstrate remarkable whiteness, with a score of 88.9, confirming their numerous benefits in terms of environmental friendliness, sustainability, analytical efficiency, and financial and practical concerns, in contrast to chromatographic reported methods [23, 26] (Fig. 9 and Table 5).

### Statistical analysis

A one-way ANOVA was carried out at a 5% significance level on the recovery % gathered from the three suggested methods and the published HPLC method [23] for the pharmaceutical dosage form (Table 6). The outcomes revealed no noteworthy differences (P > 0.05) among the techniques. Thus, the described methods are considered appropriate for precisely quantifying OLA, FLU, and FMP in their ternary mixtures and pharmaceutical formulations.

**Table 6:** statistical analysis using one-way ANOVA with a 95% confidence interval on the recovery percentage findings from the three suggested techniques and the published method [23] on pharmaceutical preparation

One way ANOVA dependent variable: recovery percentage data						
Source of variation	SS	df^a^	MS	F^b^	P-value	F crit^d^
OLA						
Between groups ^c^	3.942	3	1.314	1.825	0.221	4.066
Within groups	5.762	8	0.720			
Total	9.704	11				
FLU						
Between groups ^c^	3.163	3	1.054	3.869	0.056	4.066
Within groups	2.18	8	0.273			
Total	5.343	11				

^a^Degree of freedom

^b^F is the ratio of mean square to error mean square

^c^Between the proposed methods (the dual-wavelength ratio spectrum, PLS, and ANNs) and published HPLC method [23]

^d^The tabulated value of F

## Conclusion

New UV spectrophotometry techniques employing green solvents and supported by both univariate and chemometric methods are considered straightforward, reliable, and environmentally friendly, in contrast to the chromatographic approaches discussed here, which involve extensive use of hazardous organic solvents, complex sample handling, excessive energy usage, and reliance on advanced, high-cost instruments. The greenness and whiteness evaluations were conducted via NEMI, ESA, complex GAPI, AGREE, and RGB 12, all of which yielded better results than chromatographic methods. Chemometric methods have shown superiority over univariate techniques, requiring less time and involving fewer steps. They can detect lower concentrations within the linear range and effectively quantify impurity concentrations. An important emphasis was the integration of the sophisticated statistical pattern referred to as LHS to create an ideal validation dataset. LHS facilitates a rigorous and impartial evaluation of the model’s generalization ability throughout the whole concentration range, addressing a common limitation in chemometrics where random data splitting is often employed. By improving predictive accuracy while using a reduced number of validation samples, this approach aligns with the fundamentals of green analytical practices. This study suggests a path for analytical progress that is environmentally friendly, customized to specific needs, and focused on value, thereby making a significant contribution to sustainable development goals.

## Supplementary information

Supplementary Material 1

## Data Availability

Upon reasonable request, the corresponding author will provide the datasets used and/or analyzed in this study. The datasets utilized and/or analyzed in this study can be obtained from the corresponding author upon reasonable request.
