# Supplementary material for: Spectrophotometric determination of olanzapine, fluoxetine HCL and its impurity using univariate and chemometrics methods reinforced by latin hypercube sampling: Validation and eco-friendliness assessments
Source: BMC Chem. 2024 Oct 17;18(1):201. doi: 10.1186/s13065-024-01310-3 (PMC11484198; doi:10.1186/s13065-024-01310-3)
Supplement: Supplementary file 1 — Supplementary Material 1 [file 13065_2024_1310_MOESM1_ESM.rtf]

Fig. 1S. Show Calibration curves of FLU (5-15-20-25-35-50) ìg/ml


Fig. 2S. Shows Calibration curves of OLA (4-8-10-15-20) ìg/ml.


Fig. 3S. Typical NEMI pictograms.


PPs are assigned to each of hazard category posed by a reagent. Each reagent can have more than one PPs are assigned to each of hazard category posed by a reagent. Each reagent can have more than one hazard category, thus the sub-total PP value for a single reagent may be greater than 2.

Fig. 4S. The penalty points (PPs) to calculate the analytical Eco-Scale.


Fig. 5S. The ComplexGAPI pictogram, with the original GAPI pictogram greyed out in the background, and particular fields of the added hexagonal glyph grouped and colour-coded for clarity.


Fig. 6S. Analytical Greenness report sheets for the suggested methods by AGREE tool
